# Transcatheter closure of a residual shunt with posteroinferior deficient rim after surgical closure of an ASD: a case report

**DOI:** 10.1186/s12872-020-01624-9

**Published:** 2020-07-22

**Authors:** Xicheng Deng, Taoyue Yao, Yefeng Wang, Guangxian Yang, Wenjuan Chen, Peng Huang, Zhi Chen

**Affiliations:** 1 Heart Center, Hunan Children’s Hospital, Changsha, 410007 China; 2 The Department of Ultrasound and Echocardiography, Hunan Children’s Hospital, Changsha, 410007 China

**Keywords:** Case report, Atrial septal defect, Residual shunt, Interventional, Postoperative

## Abstract

**Background:**

There are few reports in the literature of device closure of residual shunts following initial surgical closure of an atrial septal defect (ASD). This case study reports one such case. We describe here a case of secundum type ASD that was initially closed surgically, followed by device closure of a residual shunt with a posteroinferior deficient rim.

**Case presentation:**

A 7-month-old boy was admitted to our hospital for elective surgery to surgically correct a secundum type ASD. Unfortunately, a residual shunt 3.5 mm in diameter appeared before discharge and was enlarged at1-year follow-up. The cause of this residual shunt was dehiscence at the posteroinferior aspect, and the posteroinferior rim was 3.7 mm. After careful discussion and preparation, we proceeded with an interventional procedure. A 16 mm ASD occluder (AGA Medical Corp, Plymouth, Minnesota) was deployed successfully with no residual shunt. In some cases of ASD, interventional therapy is not considered due to the size and position of the defect, but we show here, a successful case of interventional therapy for a residual shunt with a deficient rim.

**Conclusion:**

We have presented a case in which a postoperative residual shunt with a deficient rim was successfully closed with interventional therapy.

## Background

Secundum type atrial septal defect (ASD) is a common congenital heart defect [1] and interventional device occlusion is the treatment of choice as techniques and devices for transcatheter treatment have been evolved and refined [2–4]. Though generally it is acknowledged in children, transcatheter closure should be reserved for patients older than 2 years and with weight > 15 kg [5, 6], studies have shown less than 10 kg is also safe and effective in experienced hands with excellent early results [7, 8]. The alternative to transcatheter treatment is surgical repair with an antilogous pericardial or synthetic patch. Reports have been published with regard to residual shunts after surgical or interventional therapy and their treatment [9, 10]. Most residual shunts have sufficient rims to be closed with an occluder and few reports device closure of residual shunts following initial surgical closure of an atrial septal defect (ASD).

## Case presentation

A 7-month-old boy weighing 7 kg was admitted to our hospital for elective surgery to correct a secundum type ASD. The defect had been suspected during physical examination for pneumonia and was confirmed with an echocardiography previously at 4-month-old. Though he had been generally well and without tachypnea, tachycardia or cyanosis, etc., the pneumonia could not be ruled out to have had an association with this large ASD. The patient’s past medical and surgical histories were non-significant. The boy’s nutritional status and physical development were normal. He was born via C-section at 36 weeksplus1day gestation weighing 2.4 kg. While the mother experienced low amniotic fluid during pregnancy, there were no signs of intrauterine distress or asphyxia during pregnancy and delivery.

The patient’s cardiac status on admission was assessed as New York Heart Association Class II (mild symptoms). Respiratory rate was 28 breaths per minute, heart rate was122 beats per minute with sinus rhythm, and blood pressure was10.8/5.2 kPa. On auscultation, a II/6 gentle systolic murmur was heard at the left sternal border between the second and third intercostal space.

**Fig. 1:**
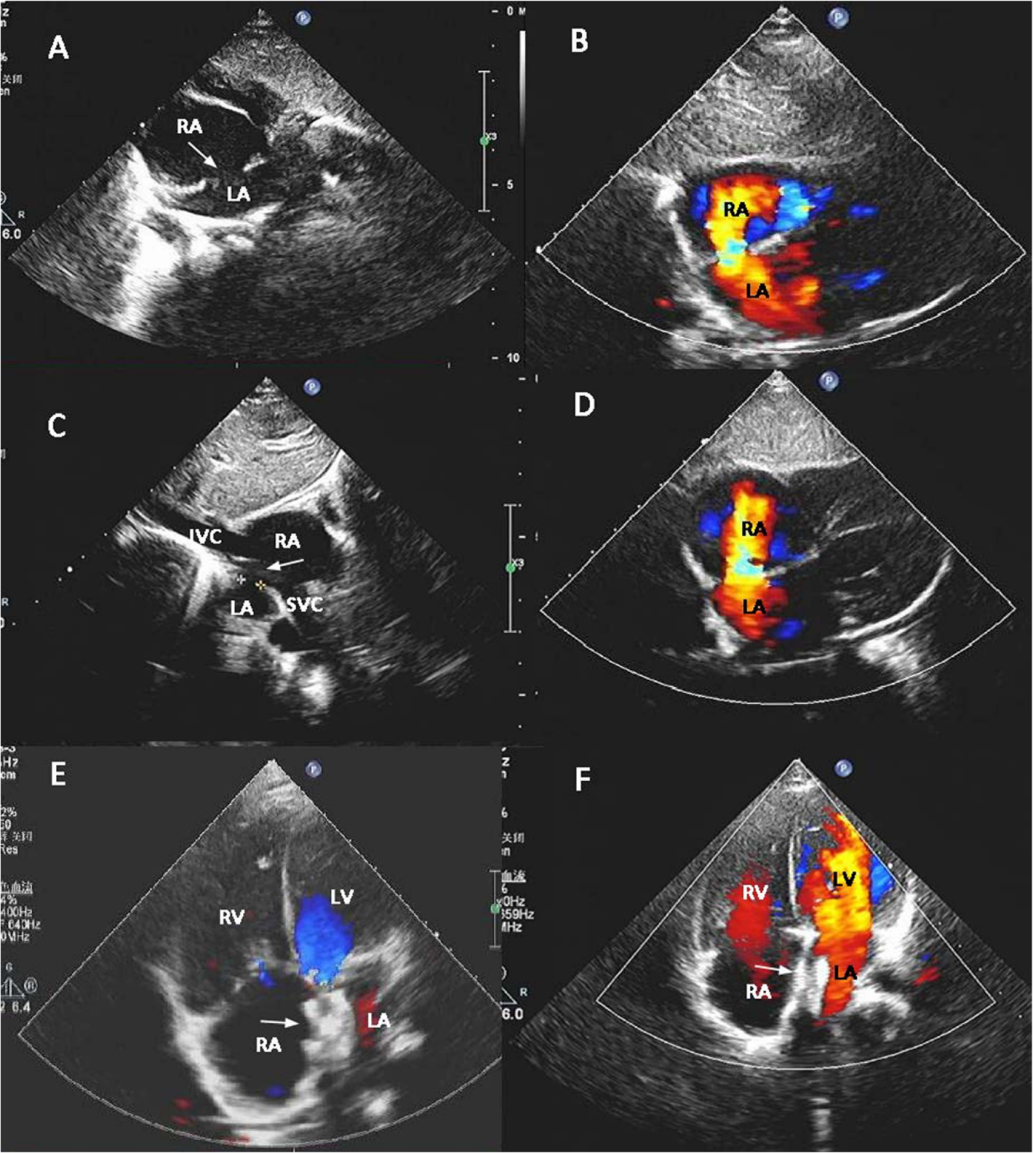
**a** Preoperative echocardiography shows the ASD, the arrow showing the defect; **b** The residual shunt 1 month after initial surgery. The red circle indicates the deficient posteroinferior rim; **c** Bicaval view of the residual shunt 3 months after initial surgery, the arrow showing the defect with minimum rim in the posteroinferior aspect; **d** The residual shunt 1 year after initial surgery; **e** Device closure of the residual shunt, the arrow showing the occluder. LA = left atrium; LV = left ventricle; **f** Three months after device closure of the residual shunt, the arrow showing the occluder. LA = left atrium, LV = left ventricle, A = right atrium, RV = right ventricle, IVC = posteroinferior vena cava, SVC = superior vena cava

Blood tests indicated slight microcytic hypochromic anemia (mean corpuscular volume 76.0 fL, hemoglobin 108.0 g/L, and mildly elevated aspartate aminotransferase 58.5 IU/L. Other values were normal. A preoperative echocardiography suggested the presence of a 15 mm by 10 mm secundum ASD (Fig. 1a), deemed quite large for a 7 months old baby. With only a 3.7 mm rim in the posteroinferior aspect, which is generally considered not amenable to cath-lab procedure. And the right heart was also shown enlarged significantly under echocardiography. Though we do not routinely measure Qp:Qs with echocardiography or perform a catheter procedure for an ASD to measure it, the signs under echocardiography indicated surgery as necessary.

At our institution, 600 open-heart cases per year were performed. Our surgeons are experienced with and confident in infants or even neonate with complex defects, so ASD repair within infancy dose not bring higher mortality or morbidity. The rationale of repair a large ASD is to avoid negative impact on growth and development that may occur if surgery is refrained until beyond infancy. Elective open-heart surgery was performed, and it was confirmed during surgery that the posteroinferior rim was insufficient for cath-lab procedure. The defect was therefore closed with an autologous pericardial patch; the procedure was straightforward. Transesophageal echocardiography confirmed that there was no residual shunt through the defect before the patient was weaned from cardiopulmonary bypass. However, the pre-discharge echocardiogram on postoperative day 5 suggested a3.5 mm-diameter shunt in the middle portion of the interatrial septum. Since it was small, follow-up was suggested. Follow-up echocardiograms at 1 month and 3 months showed the shunt to be 5–6 mm in diameter, and showed slight right cardiomegaly (Fig. 1b & c).

**Fig. 2:**
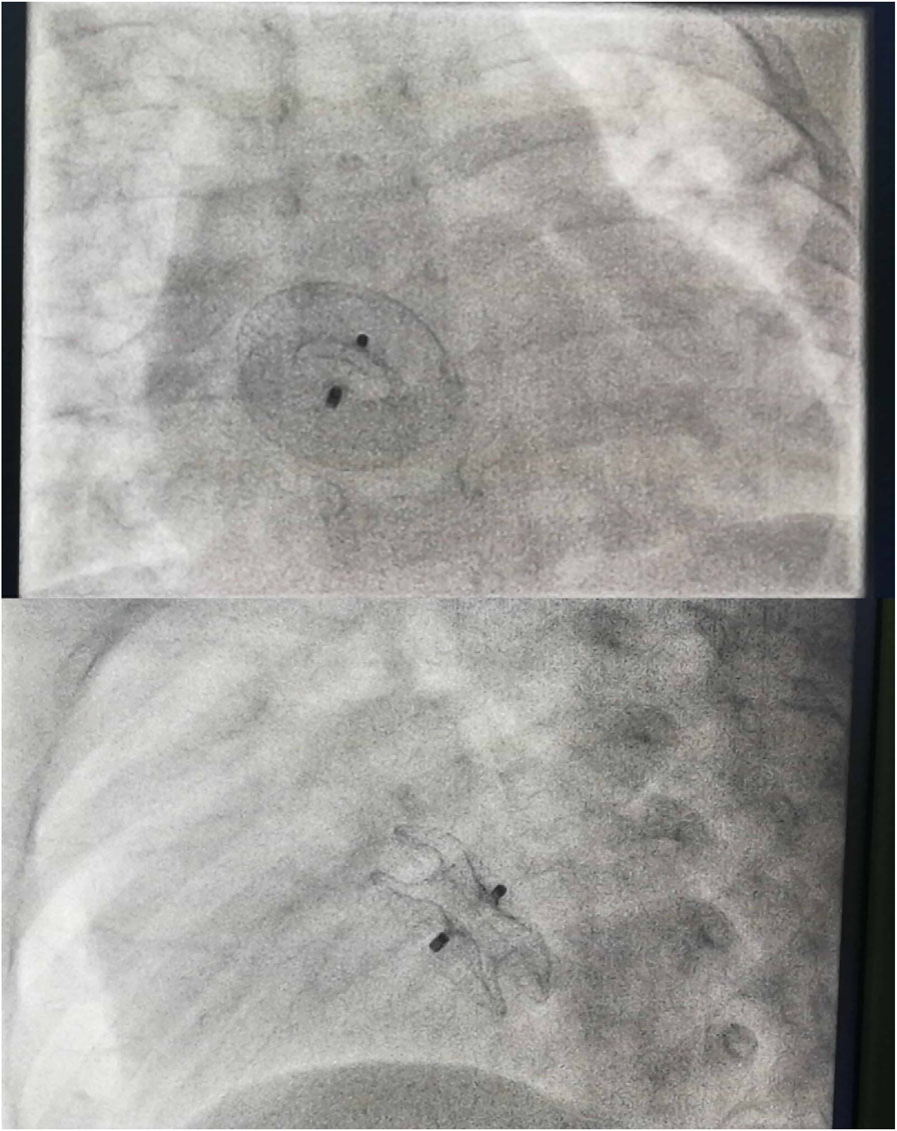
Front (top) and lateral (bottom) views after the occluder was deployed under angiocardiogram

At 1-year follow-up, when the patient was 1 year and 7 months old and weighed 10 kg, an echocardiography suggested the shunt was further enlarged to 8 mm, and the right cardiomegaly had become moderate (Fig. 1d). After careful discussion and preparation, we proceeded with an interventional procedure, with open-heart surgery planned as a backup. An additional echocardiography in the cardiac catheterization lab revealed two residual shunts of 9 mm and 4 mm diameter, and the pulmonary and systemic blood flow ratio was 1.8 as measured by right cardiac catheterization. A16 mm ASD occluder (AGA Medical Corp, Plymouth, Minnesota) was deployed successfully without any residual shunt (Figs. 1e & 2).

The 3-month postoperative follow-up showed that the patient was doing well, with the occluder in its proper position and no sign of any residual shunt under echocardiography (Fig. 1f).

## Discussion

Atrial septal defect is a common type of congenital heart disease, of which the secundum type is the most common. Cath-lab procedure with an occluder to close the defect is the standard treatment, provided there are sufficient rims around the defect and the sizes of the defects are appropriate [11]. If the rim is less than 5 mm in any aspect, it is deemed deficient and cath-lab procedure is not recommended [12], as rim deficiency is associated with significantly lower success rates [13, 14]. In this case, open-heart surgery and surgical repair of the defect may have to be chosen. Mortality and morbidity are low in the treatment of this condition, regardless of which approach is chosen [15, 16]. Some publications have reported a second device closure for residual shunt following initial transcatheter closure [10, 17, 18] or surgical treatment for residual shunt after transcatheter closure [19]. However, reports of cath-lab procedure to close a residual shunt following initial surgical closure of ASD are rare [20, 21].

In our case, open-heart surgery was performed because the posteroinferior rim, at 3.7 mm, was insufficient for transcatheter closure to be considered feasible. Since the intraoperative transesophageal echocardiography showed no residual shunt, but a shunt was detected on postoperative day 5 before discharge, it was strongly suspected that the newly detected shunt was due to suture dehiscence. For a defect with a deficient posteroinferior rim, the most likely site for dehiscence was close to the posteroinferior rim [22, 23]. This was confirmed by the final echocardiography at 1-year follow-up before residual shunt closure. The same echocardiography confirmed firm attachment of the patch to the atrial septum, with no apparent free movement of the patch. Cath-lab procedure to close the residual shunt with an occluder was planned. However, concerns remained about whether the tissue around the residual shunt was strong enough to hold an occluder in place. Moreover, the dehisced site was the posteroinferior aspect, which had been expected to have deficient rims. As such, we prepared for open-heart surgery as a backup. Our case has shown a residual shunt after surgery can be device-closed successfully even with a deficient rim in the posteroinferior aspect. This approach might be appropriate for other similar cases, including where a shunt has deficient rims in other aspects. Meticulous echocardiography examination should be carried out before any procedure. And a three-dimensional echocardiography may assist in decision-making or during transcatheter closure [24–27] in such a case.

We should, however, carefully interpret this case. As the residual shunt was smaller than the initial size of the defect and they had different configuration in terms of the aspects of the rim, the residual defect being device-closed successfully does not necessarily mean initially the ASD could be device-closed for sure, not even considering the lower weight in the initial operation. If we had the chance to do it again, we may try in a one-stop way to do it percutaneously with open heart surgery as a back-up. This should be discussed in the cardiac team on a case by case basis, accounting for patient’s age, weight and size of the defect.

## Conclusions

We have presented a case of a postoperative residual shunt with deficient rims being successfully closed with cath-lab procedure, which, in general, is considered not possible. Appropriate choice of treatment for a residual ASD should be carefully considered on a case-by-case basis after meticulous examination of a patient.

## Data Availability

All data generated or analyzed during this study are included in this published article.

## Abbreviation

ASD: Atrial septal defect
